# Development of Magnetorheological Resistive Exercise Device for Rowing Machine

**DOI:** 10.1155/2016/8979070

**Published:** 2016-05-18

**Authors:** Vytautas Grigas, Anatolijus Šulginas, Pranas Žiliukas

**Affiliations:** Mechanical Engineering Department, Mechanical Engineering and Design Faculty, Kaunas University of Technology, Studentu 56-302, LT-54214 Kaunas, Lithuania

## Abstract

Training equipment used by professional sportsmen has a great impact on their sport performance. Most universal exercisers may help only to improve the general physical condition due to the specific kinematics and peculiar resistance generated by their loading units. Training of effective techniques and learning of psychomotor skills are possible only when exercisers conform to the movements and resistance typical for particular sports kinematically and dynamically. Methodology of developing a magnetorheological resistive exercise device for generating the desired law of passive resistance force and its application in a lever-type rowing machine are described in the paper. The structural parameters of a controllable hydraulic cylinder type device were found by means of the computational fluid dynamics simulation performed by ANSYS CFX software. Parameters describing the magnetorheological fluid as non-Newtonian were determined by combining numerical and experimental research of the resistance force generated by the original magnetorheological damper. A structural scheme of the device control system was developed and the variation of the strength of magnetic field that affects the magnetorheological fluid circulating in the device was determined, ensuring a variation of the resistance force on the oar handle adequate for the resistance that occurs during a real boat rowing stroke.

## 1. Introduction

A lot of universal technical equipment, such as rubber bands and dumbbells or computerized exercising machines, is commonly used for training general physical condition (strength and endurance). In order to train/improve such specific features as the technique and coordination of movements or psychomotor skills, special exercisers should be employed. In this case, not only the nominal size of the resistance to the athlete's movements set prior to exercising, but also the kinematics of movements and variation of the resistance force during the stroke are important. These aspects of training are considered in specialized exercisers, simulating movements and loads that are specific to the particular sports (rowing, e.g.) and for rehabilitation purposes or testing the physical condition by means of dynamometers ensuring special modes like isokinetic, isotonic, and so forth [1–3]. Such special modes may be realized only by sophisticated and expensive computer-controlled electromechanical or electromagnetical devices, such as isokinetic dynamometers. Therefore, the vast majority of exercisers can only ensure a possibility to choose the nominal resistance level before starting to exercise (e.g., by selecting the proper size of the weight stack, stiffness of the rubber band, or the resistance level of the adjustable hydraulic cylinder type dampers). As a result, the variation of the resistance force during the exercise cycle depends on the kinematics of the exerciser and the athlete's efforts, the acceleration, range, and speed of movements.

The same may be said about rowing machines, especially popular due to their versatility: rowing has a significant effect on training the strength of most of the human muscles, improves endurance, and has a positive impact on cardiovascular, respiratory systems, and so forth; thus, rowing simulators are often used as ergometers by both amateur and professional rowers. While exercising, proper movement technique is especially important for the latter, as well as other professional sportsmen aiming at highest sports results [4–6]. However, due to specific features related to seasonal changes, rowing is either not always possible or quite expensive under real conditions, so attempts are continuously made to develop a rowing machine able to simulate real rowing adequately, including kinematics of movements and variation of resistance force which is specific within one stroke cycle and changes during the competition [7–9]. Hydrodynamic resistance force, characteristic to rowing, differs significantly from gravitational, inertial, or elastic resistance that is typical for most of the common exercisers, because it reaches its maximal value at the middle of the stroke. Therefore, in order to improve the rowers' training possibilities, a lot of specific hydrodynamic, aerodynamic, magnetic, or combined (with inertial) resistance rowing machines were built [10], including whole boats mounted in pools [11]. But usually they either differ significantly from the real boat by their kinematics and resistance force or are highly expensive and nontransportable.

Recent achievements in the development of smart material technologies (including fluids with controllable properties, like magnetorheological fluids (MR fluids)) have led to the creation and widening spread of technical solutions that ensure controlling the kinematics and dynamics of the various purpose machines during their operation. For instance, the magnetorheological dampers became almost a standard component of automotive suspensions and some sorts of industrial equipment. Nowadays, magnetorheological devices are also encountered in rotary-movement active or passive orthoses [12], loading units of specialized arms or legs, as well as universal training machines [13–17], and other human-powered equipment due to their rapidity, safety, reliability, and relative simplicity.

The aim of the research was to develop a resistive exercise device based on magnetorheological fluid technology and its control system for the rowing machine. The device should ensure variation of the resistance force on the oar handle adequate for hydrodynamic resistance which occurs during a real boat rowing stroke.

## 2. Structure and Main Properties of the Lever-Type Rowing Machine

### 2.1. Structure of the Lever-Type Rowing Machine

Among a great variety of rowing machines, lever-type simulators, such as “Kettler Kadett” [18] or “HAMMER Cobra” [19] equipped with linear hydraulic cylinder (HC) type load generating units, are the most adequate for a real sculling boat. The two main issues making these simulators most useful are the kinematics of the levers corresponding to boat oars (making the athlete's movements very similar to the real rowing) and the hydrodynamic character of the resistance force (the resistance during the exercise cycle depending on how fast the oar is pulled). Although the geometry of the mentioned exercisers (lengths of the levers, position of hinges/oars locks, etc.) is not exactly the same as in real boats, differences are relatively small and may be easily evaluated during further improvement of the device. In addition, the relative simplicity of such a rowing machine design implies the possibility of replacement of regular hydraulic cylinders by controllable (during single strokes and their sequence) resistive exercise devices.

**Figure 1:**
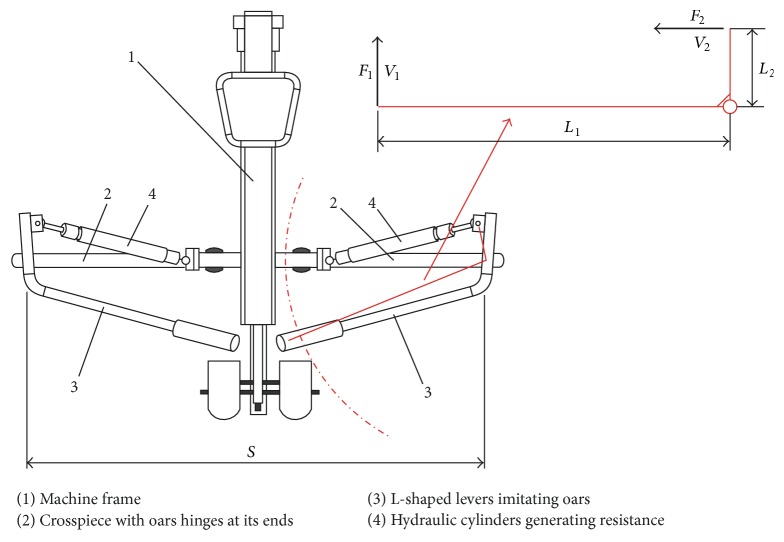
Structure of lever-type rowing machine and kinematical scheme of resistance system: *S*: spread (scull), *L*_1_ and *L*_2_: lengths of L-shaped levers imitating oar handles (*S* = 1.58 m, *L*_1_ = 0.74 m, and *L*_2_ = 0.135 m), *F*_1_: force on oar handle, and *F*_2_: resistance force generated by hydraulic cylinder.

**Figure 2:**
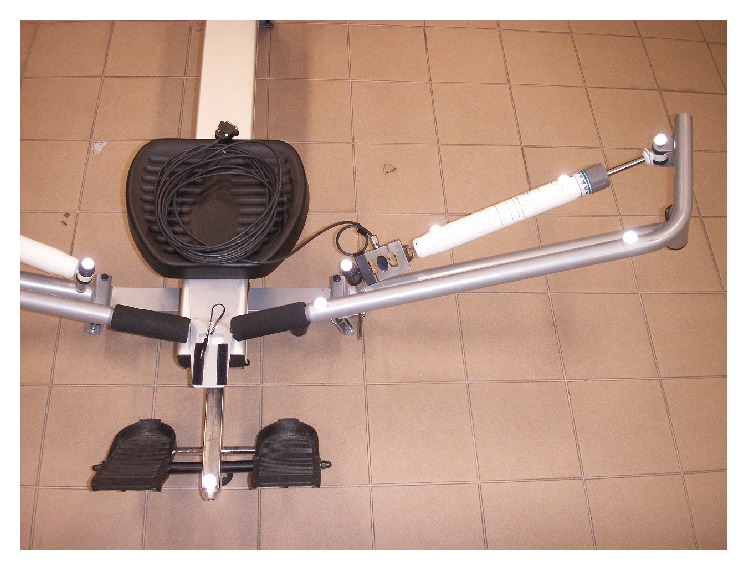
Location of the markers of 3D Qualisys mocap system on the rowing machine and HBM S9 type force sensor inserted between the hydraulic cylinder bottom end and the frame.

The lever-type rowing machine “HAMMER Cobra” [19] equipped with manually adjustable resistance hydraulic cylinders (12 levels: 1st, the lowest, and 12th, the highest, defined by adjusting the ring on the rod end of the cylinder) was used as a prototype (Figures 1 and 2). The main geometrical parameters of the rowing machine (Figure 1), determining the degree of its kinematic similarity to a real boat, are the spread (scull) or the distance between oars hinges/locks *S* (1.58 m) and the lengths of the levers representing oar handles *L*_1_ (0.74 m) having the arms *L*_2_ (0.135 m), to which the hydraulic cylinders rods are connected (arms' ratio *L*_1_/*L*_2_ = 5.5).

### 2.2. Kinematic-Dynamic Properties of the Lever-Type Rowing Machine

Kinematic characteristics of the rowing machine (movement of the levers simulating oar handles and piston rods of hydraulic cylinders) were measured by using 3D Motion Capture system “Qualisys” controlled by “QTM” (“Qualisys Track Manager”) software (Qualisys, Sweden). The control points (cylinder and rod ends, ends of levers, and centers of hinges connecting them) were marked by 15 mm diameter spherical reflective markers (Figure 2), and the variation of their positions during exercising was recorded in real time mode by six computer-controlled 500 Hz frequency infrared spectrum digital cameras. Resistance force *F*_2_, generated by the HC type load generating unit, was measured simultaneously by means of the mobile multichannel system “Spider Mobil-8” (HBM, Germany) with the 2500 N capacity S9 type force sensor inserted between the hydraulic cylinder cap end and the frame (Figure 2).

In this way, the dependency of resistance force *F*_2_ generated by HC type load generating unit on the HC piston displacement and HC rod/piston displacement dependency on time (thereby its speed) during the rod extraction caused by pulling the levers imitating oars handles was determined. An experienced youth female rower (aged 22), practicing actively for more than 5 years, took part in the investigation. Warm-up was made at the beginning of each measurement; then measurements were performed with different resistance levels of the cylinder (1st, 5th, and 9th) set before exercising and at two different rates (normal and fast: 24 and 30 cycles per minute, resp.), not less than 10 strokes for each combination of parameters.

The results of the investigation of the rowing machine kinematics and resistance force were compared with the results of experimental research carried out earlier by the authors of this paper [20, 21] or described in [10, 22–24].

The measurements of the kinematical parameters of the rowing machine have shown that the stroke of the hydraulic cylinder approximately equals 170 mm and practically does not depend on the selected resistance level and exercising rate. The maximal speed of HC rod extraction at the lowest (1st) resistance level is 0.22 m/s when exercising at a normal rate and near 0.3 m/s when exercising at a fast rate (in the recovery phase 0.3 m/s and 0.35 m/s, resp.). Research of the rowing boat fixed in the pool, described in [21], gave the speed of oar handle 1.5 m/s in the drive phase and 2 m/s in the recovery phase. For a rowing machine having the L-shaped lever arms *L*_1_ and *L*_2_ ratio equal to 5.5, these parameters would correspond to 0.27 and 0.36 m/s speed of the rod/piston insertion, respectively.

**Figure 3:**
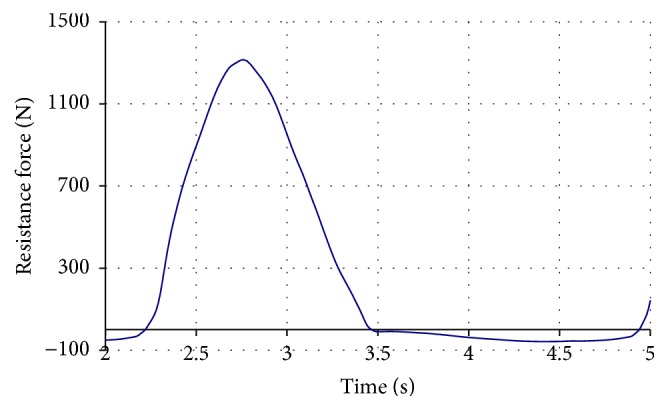
The law of change of resistance force generated by hydraulic cylinder type load generating unit of HAMMER Cobra rowing machine during a single stroke cycle (exercising rate 24 strokes/min, resistance level: 5).

The measurements of the HC resistance have shown that in the drive phase of the stroke the law of change of the resistance force generated by the adjustable hydraulic cylinder of the “HAMMER Cobra” rowing machine (Figure 3) is of similar hydrodynamic character as the resistance force of the most popular rowing simulators Concept II [10] or Rowperfect [25]. However, it is not exactly the same as obtained by measuring the rowing force when rowing a real boat [20, 23, 24] inter alia depending on the position of the rower in a boat, the stage of the race, and other factors [26]. The drive force impulse curve is very smooth and almost symmetrical; resistance in the recovery phase is near to zero (dozens of newtons). It should also be noted that, due to the peculiarities of such type rowing simulator design, the resistance force practically does not depend on the vertical position of the oar handle. In reality, it corresponds to the depth of immersion of the oar blade into water, which means that, from this point of view, the lever-type rowing machine does not meet real rowing conditions.

In the case of real rowing, the force on the oar handle was about 600 N in the drive phase and 100 N in the recovery phase (male athletes) [20]. The maximal resistance force generated by the hydraulic cylinder load generating unit (female athlete) was about 1100 N at the first resistance level and 1250 N at the fifth resistance level. When the ninth resistance level was set, the attempts to exercise failed due to too high resistance force exceeding 1800 N (at a lower exercising rate). If these forces are recalculated to the force on oar handle and the ratio of arms *L*_1_ and *L*_2_ of the L-shaped lever of the rowing machine is considered, 5.5 times lower resistance will be received: 210–330 N peaks in the middle of the drive phase and 10–20 N almost during the whole recovery phase (i.e., 2-3 times smaller than in the referenced case).

### 2.3. Resistance Force Generated by Linear Stroke Hydraulic Cylinder Type Load Generating Units

In order to collect more information about functionality of the HC type load generating units used in lever-type rowing machines, experimental research of their resistance force was carried out [27]. The variation of resistance force size during the stroke of adjustable hydraulic cylinders taken from three rowing machines was measured at different operating modes: the speed of rod extraction, resistance level of the cylinder, and the temperature of the cylinder. All the three cylinders were of the same twin-tube design; the diameter of their pistons was 25 mm, external diameter 65 mm, and stroke ~275 mm. Investigation showed that force characteristics of all the three adjustable HCs apparently differ both qualitatively and quantitatively: the force curves are quite uneven (variation of the resistance force is up to 30% of the nominal value at constant velocity of the piston) and the nominal resistance force size is different even when the same resistance level (1st–12th) is set before exercising. Thus, even if the design and kinematics of all rowing machines seem to be very similar, the resistance on the oar handle may be quite different for different exercisers even at the same resistance level set by the adjustment ring. Therefore, it is important to use a proper cylinder when replacing either a broken or a worn one, or both cylinders should be replaced to ensure the symmetry of resistance.

**Figure 4:**
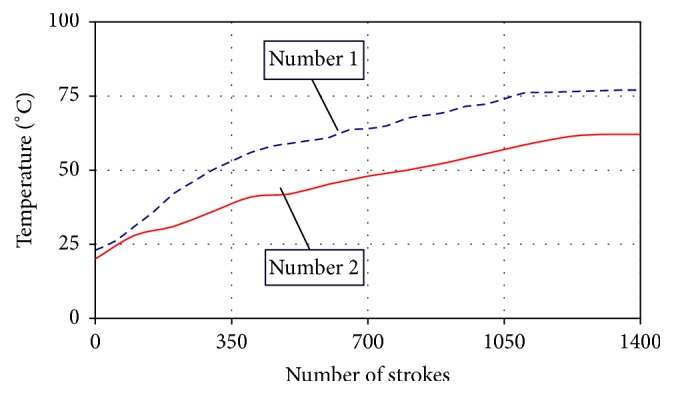
Dependencies of the temperature of hydraulic cylinder numbers 1 and 2 on the number of exercise cycles (exercising rate: 50 strokes/min).

**Figure 5:**
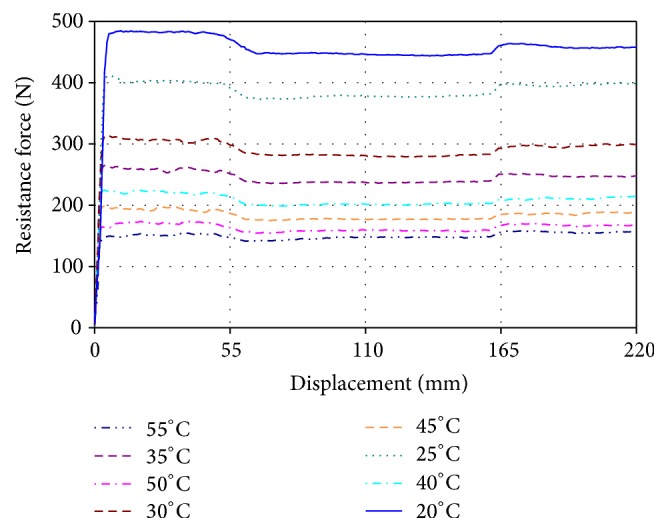
Dependencies of the resistance force generated by hydraulic cylinder number 2 on its body temperature (speed of rod/piston extraction: 0.015 m/s).

In parallel with the research described above, the dependency of resistance force of the HC type load generating units on their body temperature has been investigated, taking into account its evident rise during exercising. The resistance characteristics and the temperature of the cylinder body were measured every 100 cycles of exercising by means of a universal computerized two-column desktop machine for testing materials' mechanical properties “H25KT” (Tinius Olsen, USA) equipped with the force sensor HTE-1000 N and controlled by the “QMAT” software. HC body temperature was measured by an infrared (IR) thermometer “Testo 845” (Testo, Inc., USA). When rowing at the rate of 50 strokes/min, the temperature of hydraulic cylinder number 1 body reached 76°C after 1400 strokes and the temperature of hydraulic cylinder number 2 63°C (Figure 4), which caused a corresponding drop of the resistance force. For example, the resistance force of hydraulic cylinder number 1 decreased from 450 N at 20°C temperature down to 150 N as it heated up to 55°C (Figure 5). These facts allow us to state that, even at relatively low exercising intensity, the resistance level of the hydraulic cylinder should be periodically adjusted (increased) in order to maintain constant resistance during the whole exercising process.

Thus, the study of a rowing machine, real rowing kinematics, and the resistance force gave the information about the range and characteristics of the resistance force and HC piston/rod speed that should be ensured to make the law of resistance force adequate for real rowing.

The research of dynamic characteristics of HC type load generating units used in lever-type rowing machines also allows us to conclude that several essential deficiencies are typical to such devices (and the machines where they are used):

- Stability of the resistance force at a constant speed of the rod/piston extraction is not ensured.
- The size of resistance force depends significantly on the HC temperature increasing with the number of exercise cycles that requires periodical adjustment of the load generating unit during exercising.
- The similarity of the law of resistance force variation to the resistance obtained during real rowing is only qualitative; some factors (like depth of oar immersion into water) are neglected.
- There is no possibility to control the resistance force during an exercise cycle.

To eliminate the above-mentioned deficiencies of regular lever-type rowing machines equipped with the (manually) adjustable HC type load generating units, these units should be replaced by controllable (during stroke or sequence of strokes) resistive exercise devices, for example, based on magnetorheological fluid technology.

## 3. Methods of Solution

To improve a rowing machine so that it would better correspond to real rowing, it is enough to replace its regular HC type load generating units by controllable (during stroke) resistive exercise devices. Such devices should generate resistance dependent not only on the exercising rate and consequently the speed of pulling oar handles, but also on various kinematic-dynamic parameters characterizing the run of each rowing stroke or even the sequence of strokes (if simulation of different training modes or competition is desired). These parameters should be measured by the set of sensors (vertical and horizontal position and angular velocities of levers simulating oars, resistance force, etc.) integrated with a programmable control system adjusting the resistive exercise devices. The magnetorheological fluid technology seems to be most easily implemented for this type of machinery with such functional parameters. The control of resistance force generated by such a device is ensured by controlling the viscosity of the MR fluid circulating within it by means of a magnetic field. The more viscous the fluid, the larger the resistance to its flow, what in the case of a hydraulic cylinder type device, where the fluid flows between the chambers separated by the movable piston, may be related to the speed of piston/rod insertion/extraction under the effect of external force.

Development of such peculiar devices is hardly imaginable without application of computer aided technologies, namely, computational fluid dynamic (CFD), especially in the case of application of smart materials, such as MR fluids, having very specific material properties. Thus the first problem that arose after the decision was made to develop a hydraulic cylinder type controllable resistive exercise device based on MR fluid technology, proper description of such fluid properties being definitely non-Newtonian. It is related to the lack of information about dependencies of commercially available MR fluid properties on the strength of the magnetic field. After this problem was solved, as described in Section 3.1, the magnetorheological resistive exercise device was designed and its parameters ensuring the necessary range of the resistance force were determined, as shown in Section 3.2. Finally, the law of change of the magnetic field acting on the MR fluid within the magnetorheological resistive exercise device was derived, which ensures the law of variation of the resistance force adequate for the resistance force on the oar handle when rowing a real boat (Section 3.4).

### 3.1. Parameters of Non-Newtonian Model of the Magnetorheological Fluid

MR fluids are non-Newtonian; that is, their relationship between the shear stress and shear strain rate is not simply linear. ANSYS CFX software used during this research has several models for calculating viscosity based on shear strain rate [28]. Among them, the Herschel-Bulkley mathematical model for viscoplastic fluids appears to be the most suitable when the MR fluid MRF140-CG (Lord, USA [29]) is applied in the device to be designed [30]. To describe the dynamical viscosity of such fluids by this mathematical model, two specific parameters are required: viscosity consistency *K* and yield stress *τ*_*Y*_ of the fluid, both dependent on the strength of the magnetic field acting on the fluid:

(1) $$\begin{array}{c} \mu =\frac{\tau_{Y}}{\left(\lambda \dot{\gamma }\right)}+K\cdot {\left(\;\lambda \dot{\gamma }\;\right)}^{n-1}, \end{array}$$

where *K* is viscosity consistency (Pa·s^*n*^) defining viscosity of a fluid, *n* is linear power law index of the fluid, *τ*_*Y*_ is yield stress of the fluid (Pa), $\dot{\gamma }$ is gradient of shear velocity (or shear strain rate) (1/s), and *λ* is time constant.

The yield stress *τ*_*Y*_ dependency on the strength of the magnetic field for MR fluid MRF140-CG is presented by its manufacturer [29], while the effect of the magnetic field on viscosity consistency *K* is not specified; thus this is the only unknown factor that should be found to describe physical properties of MR fluid adequately.

**Figure 6:**
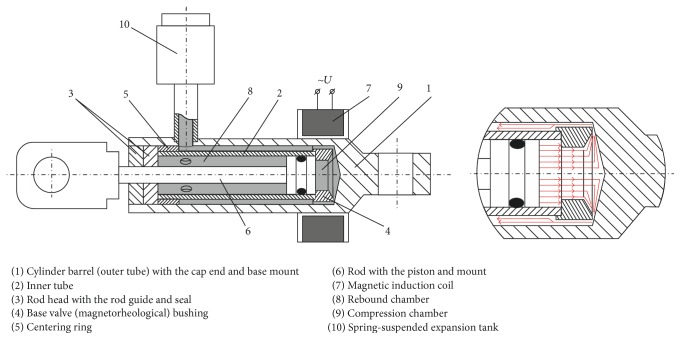
MR damper section view.

For this purpose, a computer aided simulation of an original design MR damper manufactured at KTU (Figure 6) was carried out, using the results of experimental investigation of its resistance force when operating at different modes as reference data [31]. The main structural parameters of a single-acting twin-tube hydraulic cylinder type MR damper are as follows: piston (and inner tube internal) diameter: 13 mm, inner tube external diameter: 16.5 mm, piston rod diameter: 6 mm, stroke: 44 mm, and outer tube internal/external diameters: 25.4/20 mm.

**Figure 7:**
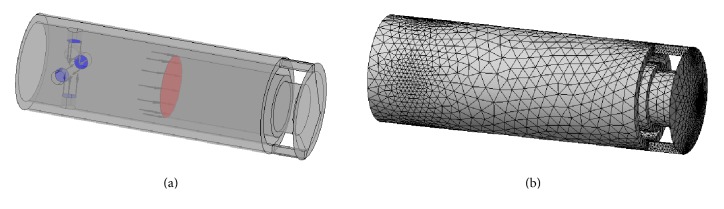
Models of MR fluid damper: (a) computational domain with boundary conditions indicated; (b) finite element mesh of computational domain (~390.000 volume elements).

At first, a 3D geometrical model of the MR damper and a computational finite element model of computational domain, corresponding to its cavities filled with MR fluid (Figure 7), were created. The finite element mesh consisted of ~390.000 0.03–6.3 mm size volume elements (the mesh was refined in the zones of outlet areas and narrowest channels of the base valve). Then the numerical simulation of MR damper operation was performed by using ANSYS CFX CFD code. During the simulation, the resistance force generated by the MR damper, resulting from the pressure acting on piston 6 moving inside cylinder 2, was found for different combinations of piston 6 speeds (0.001, 0.002, 0.035, 0.005, and 0.0075 m/s) and the strength of the magnetic field generated by magnetic coil 7 (0, 13, 25, and 38 mT), affecting the MR fluid in the zone of magnetorheological base valve 4. Computations were performed by specifying the inlet velocity of the fluid at the round surface corresponding to the end of the piston (larger circle in the middle of computational domain (Figure 7(a))) and the outlet to the nonpressurized environment via four circular openings connecting the rebound chamber inside the inner tube with the tubular channel between inner and outer tubes (four smaller circles at the left end of computational domain (Figure 7(a))). For simplification reasons, the effect of the magnetic field was evaluated by specifying corresponding values of the viscosity consistency *K* and yield stress *τ*_*Y*_ to the whole computational domain containing MR fluid, though its main effect occurs in the zone of maximal gradient of the flow velocities, namely, in the magnetorheological base valve 4. It is made as bushing with two narrow channels (1 × 1 mm^2^ cross section), where the fluid flows with maximal speed from compression chamber 9 to rebound chamber 8, thereby being affected by the magnetic field generated by magnetic coil 7 (Figure 6).

Two states of the MR fluid corresponding to the MR damper operation in passive and active modes were evaluated during the analysis. In the first case, the MR fluid MRF140-CG was described as Newtonian and in the second as non-Newtonian. The main physical properties of the MR fluid (for both Newtonian and non-Newtonian models) are presented in Table 1.

In the second case, a series of computations with different values of viscosity consistency *K* were carried out until the satisfactory adequacy of the average resistance force caused by the pressure of the MR fluid acting on the piston to the results of the experimental measurements mentioned above [31] was reached for all the three cases of the magnetic field strength and for five speeds of piston movement (or fluid inlet velocity).

### 3.2. Computational Analysis of MR Fluid Based Devices

Non-Newtonian behavior of the fluid circulating in the MR damper cavities is simulated by switching on the dynamic viscosity option in ANSYS CFX preprocessor material properties description window. Then the selection of the fluid model and viscosity model is performed, which makes it possible to specify four main parameters describing MR fluid MRF140-CG as non-Newtonian (Hershel-Bulkley type, described by (1)): power law index, shear strain rate (or shear velocity), yield stress, and viscosity consistency.

The hydrodynamic force acting on the piston is obtained by integrating fluid pressure acting on the piston end surface, which in turn is obtained by solving a typical set of unsteady Navier-Stokes equations in their conservation form (continuity, momentum, energy, pressure, and turbulence) describing a single-phase, single-domain steady state incompressible fluid flow problem. It is defined by the laws of conservation of mass, momentum, and energy, expressed in terms of partial differential equations which are discretized with a finite element based technique used in computational fluid dynamics code ANSYS CFX [32].

#### 3.2.1. Transport Equations [32]

The instantaneous equations of mass, momentum, and energy conservation can be written as follows in a stationary frame (the symbol description is the following: *U* is velocity magnitude, $\overset{-}{U}$ is vector of velocity *U*_*x*,*y*,*z*_, *c*_*p*_ is specific heat capacity at constant pressure, *h* is specific static (thermodynamic) enthalpy, *h*_tot_ is specific total enthalpy, *τ* is shear stress, *p* is static (thermodynamic) pressure, ${\overset{-}{S}}_{M}$ is momentum source, ${\overset{-}{S}}_{E}$ is energy source, *T* is static (thermodynamic) temperature, *ρ* is density, *δ* is identity matrix, *μ* is molecular (dynamic) viscosity, and *λ* is thermal conductivity).

The continuity equation is

(2) $$\begin{array}{c} \frac{\partial \rho }{\partial t}+\nabla \cdot \left(\;\rho \overset{-}{U}\;\right)=0. \end{array}$$

The momentum equation is

(3) $$\begin{array}{c} \frac{\partial \left(\rho \overset{-}{U}\right)}{\partial t}+\nabla \cdot \left(\;\rho \overset{-}{U}\times \overset{-}{U}\;\right)=-\nabla p+\nabla \cdot \tau +{\overset{-}{S}}_{M}, \end{array}$$

where the stress tensor, *τ*, is related to the strain rate by

(4) $$\begin{array}{c} \tau =\mu \left(\;\nabla \overset{-}{U}+{\left(\;\nabla \overset{-}{U}\;\right)}^{T}-\frac{2}{3}\delta \cdot \nabla \cdot \overset{-}{U}\;\right). \end{array}$$

The total energy equation is

(5) ∂ρhtot∂t−∂p∂t+∇·ρU−htot=∇·λ∇T+∇·U−·τ+U−·S−M+S−E,

where *h*_tot_ is the total enthalpy, related to the static enthalpy *h*(*T*, *p*) by

(6) $$\begin{array}{c} h_{tot}=h+\frac{1}{2}{\overset{-}{U}}^{2}. \end{array}$$

The term $\nabla \cdot (\overset{-}{U}\cdot \tau )$ represents the work due to viscous stresses and is called the viscous work term. It models the internal heating by viscosity in the fluid and is negligible in most flows. The term $\overset{-}{U}\cdot {\overset{-}{S}}_{M}$ represents the work due to external momentum sources and is currently neglected.

The thermal energy equation is

(7) $$\begin{array}{c} K=\frac{1}{2}{\overset{-}{U}}^{2}. \end{array}$$

The mechanical energy equation is derived by taking the dot product of *U* with the momentum equation (3):

(8) $$\begin{array}{c} \frac{\partial \left(\rho K\right)}{\partial t}+\nabla \cdot \left(\;\rho \overset{-}{U}K\;\right)=-\overset{-}{U}\cdot \nabla p+\overset{-}{U}\left(\;\nabla \cdot \tau \;\right)+\overset{-}{U}\cdot {\overset{-}{S}}_{M}. \end{array}$$

Subtracting this equation from the total energy equation (5) yields the thermal energy equation:

(9) ∂ρh∂t−∂p∂t+∇·ρU−h=∇·λ∇T+U−·∇p+τ:∇U−+S−E.

The term $\tau :\nabla \overset{-}{U}$ is always positive and is called the viscous dissipation. It models the internal heating by viscosity in the fluid and is negligible in most flows.

#### 3.2.2. Equation of State [32]

The transport equations described above must be augmented with constitutive equations of state for density and for enthalpy in order to form a closed system. In the most general case, these state equations have the form:

(10) ρ=ρp,T,dh=∂h∂TpdT+∂h∂TTdp,cp=cpp,T.

Incompressible equation of state is the simplest case: density is constant and *c*_*p*_ can be (at most) a function of temperature:

(11) ρ=ρspec,dh=cpdT+∂pρ,cp=cpp,T.

Specific notations used in equations above are as follows. When Cartesian coordinate system is assumed, in which *i*, *j*, and *k* are unit vectors in the three coordinate directions, vector operator ∇ is defined such that ∇ = [∂/∂*x*, ∂/∂*y*, ∂/∂*z*]. For a vector function $\overset{-}{U}(x,y,z)$, where $\overset{-}{U}=\left[\begin{array}{c} U_{x}\\ U_{y}\\ U_{z} \end{array}\right]$, the divergence of $\overset{-}{U}$ is defined by $\nabla \cdot \overset{-}{U}=\partial U_{x}/\partial x+\partial U_{y}/\partial y+\partial U_{z}/\partial z.$ The dyadic operator (or tensor product) of two vectors, $\overset{-}{U}$ and $\overset{-}{V}$, is defined as $\overset{-}{U}\times \overset{-}{V}=\left[\begin{array}{ccc} U_{x}V_{x} & U_{x}V_{y} & U_{x}V_{z}\\ U_{y}V_{x} & U_{y}V_{y} & U_{y}V_{z}\\ U_{z}V_{x} & U_{z}V_{y} & U_{z}V_{z} \end{array}\right]$. In index notation, the divergence operator can be written as $\nabla \cdot \overset{-}{U}=\partial U_{i}/\partial x_{i}$, where the summation convention is followed; that is, index *i* is summed over the three components. The quantity $\overset{-}{U}\times \overset{-}{V}$ can be represented by *U*_*i*_ *V*_*i*_ (when $\overset{-}{U}$ and $\overset{-}{V}$ are vectors) or by *U*_*i*_ *V*_*jk*_ (when $\overset{-}{U}$ is a vector and $\overset{-}{V}$ is a matrix), and so on. Hence, the quantity $\nabla \cdot (\rho \overset{-}{U}\times \overset{-}{U})$ can be represented by (∂/∂*x*_*i*_)(*ρU*_*i*_ *U*_*j*_).

Dynamic viscosity *μ*, dependent on the magnetic field strength (1), in the case under discussion, appears in the momentum equation (3) (stress tensor, *τ* (4)).

### 3.3. Magnetorheological Resistive Exercise Device for Rowing Machine

**Figure 8:**
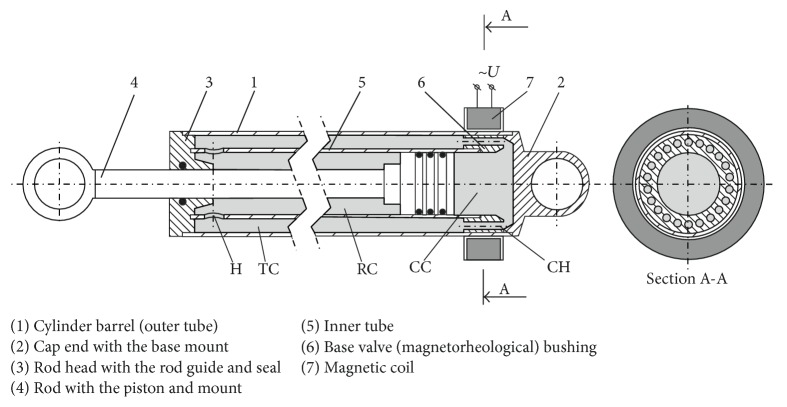
Scheme of MR resistive exercise device for rowing machine: CC: compression chamber; RC: rebound chamber; CH: circular channels in the bushing of magnetorheological base valve; TC: tubular chamber; H: radial holes connecting tubular and rebound chambers.

The structural scheme of a twin-tube linear motion magnetorheological resistive exercise device (Figure 8) for a lever-type rowing machine was developed on the base of the data obtained during experimental and computational investigation of a lever-type rowing simulator, hydraulic cylinder type load generating units, and magnetorheological damper.

The operation principle and the construction of the device are analogous to those of a MR damper. During the compression of the device (recovery phase of rowing cycle) with rod 4 initially extracted, the piston attached to the internal end of the rod pushes the MR fluid, forcing it to flow from the compression chamber CC to the tubular chamber TC between inner and outer tubes 1 and 5 through the channels CH made in the bushing of magnetorheological base valve 6. Resistance to the MR fluid flow depends on the number, length, and cross-sectional area of the channels CH and of course viscosity of the fluid, which in turn depends on the strength of the magnetic field controlled by the strength of the current in magnetic coil 7 surrounding base valve 6. Then the fluid passes from the tubular chamber TC to the rebound chamber RC through four round radial holes H in the inner tube near its rod end (the diameter of these holes is significantly larger than that of channels CH to ensure lower resistance). At the backdraught, when the rod with the piston is extracted (drive phase of rowing cycle), MR fluid travels conversely and passes the base valve in the opposite direction, thus again giving a possibility to control the process by means of the magnetic field affecting its viscosity.

**Figure 9:**
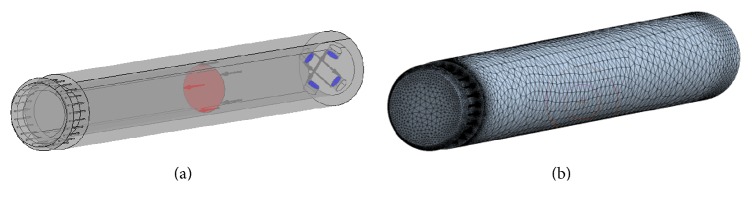
Linear motion hydraulic cylinder type MR resistive exercise device for lever-type rowing machine: (a) computational domain geometrical model with boundary conditions; (b) finite element mesh (952.000 volume elements).

Based on this scheme, the parameterized 3D geometrical and computational models (Figure 9) of the MR resistive exercise device were elaborated by using ANSYS CFX CFD code (finite element mesh consisted of ~952.000  0.03–6.3 mm size volume elements; it was refined in the zones of outlet areas and narrowest channels of the base valve). General dimensions of the device, the internal diameter of the inner cylinder (and the diameter of the piston) and the stroke, were selected to correspond to the parameters of the hydraulic cylinder used in the rowing machine “HAMMER Cobra” [19]. The remaining parameters (number, diameter, and length of the channels generating resistance to the flow) were determined by performing a series of steady state flow computations with models having different combinations of the mentioned parameters until the set of parameters ensuring the necessary range of resistance force (minimal resistance at the recovery stroke, i.e., during the rod retraction, and maximal one at the drive stroke or rod extraction) at corresponding speeds of piston displacement was found. The effect of different strength of the magnetic field affecting MR fluid MRF-140CG was taken into account during computations by using its non-Newtonian Herschel-Bulkley model characterized by the magnetic field strength dependent parameters like viscosity consistency *K* and yield stress *τ*_*Y*_ obtained during the previous stage of the research.

At first, the number and the diameter of channels in the MR valve bushing of a resistive exercise device ensuring minimal resistance force appearing during the recovery phase of the rowing cycle were based on the results of investigations of the rowing force conducted earlier and described in [20]. This analysis was made by describing MR fluid as Newtonian (main physical properties according to Table 1 (left column)) that is neglecting the effect of the magnetic field on fluid viscosity. The speed of rod/piston retraction obtained during the first stage of the research (and compared with the results of investigations carried out earlier and described in [21]) was used during these computations. The number of channels was changed from 5 to 30 every 5 and their diameter increased from 0.4 to 2 mm every 0.4 mm.

Afterwards, computations of the resistance force generated by the resistive exercise device at different speeds of the rod/piston extraction (0.1, 0.2, and 0.3 m/s) and different strength of the magnetic field (0–38 mT) were carried out to find the set of parameters (number and diameter of channels in the bushing of MR valve) ensuring the necessary maximal resistance force obtained during the first stage of the research and during the experimental investigations performed earlier [20].

### 3.4. Control of the Resistance Force within Single Rowing Stroke

The aim of the last stage of numerical simulation of a MR resistive exercise device for a lever-type rowing machine was to determine how the strength of the magnetic field controlling the MR fluid viscosity (and thus the size of the resistance force) should be changed during one rowing stroke to ensure the pattern of this force adequate for the force resulting when rowing a real boat.

To that purpose, one more series of computations was carried out using the model of resistive exercise device with the parameters that were obtained in the previous stage of the research. In the first instance, the normalized reference curve of cylinder compression speed during the rowing stroke cycle drive phase was built on the base of the results of investigations conducted earlier [21] (whichever curve may be used in the particular case) and divided into 50 steps. After that, the size of the resistance force for each of the 50 steps with a corresponding piston speed was computed at a different strength of the magnetic field (0, 13, 25, and 38 mT). Thus a variation of the resistance force during the rowing cycle at constant magnetic field of different strength was obtained. Next, the normalized reference curve of the resistance force during the rowing stroke drive phase was built on the results of investigations conducted earlier [20] (whichever curve may be used in the particular case, too). It was also divided into 50 steps and computations of the resistance force at each step were carried out by using piston speed at each step according to the cylinder compression speed reference curve mentioned above. But, in this case, the strength of the magnetic field affecting the MR fluid was changed until the size of the calculated resistance force matched the size of the force at the same step of the reference force curve. In this way, the law of change of the magnetic field strength, ensuring the necessary pattern of the resistance force during a single rowing strike, was defined.

## 4. Results and Discussion

### 4.1. Parameters of Non-Newtonian Model of the Magnetorheological Fluid

Assuming that control of resistance force generated by the resistive exercise device is performed by means of the magnetic field which influences viscosity of the MR fluid MRF140-CG circulating in the device, the dependence of viscosity consistency *K* (Herschel-Bulkley mathematical model in ANSYS CFX CFD code) on the strength of magnetic field was evaluated. It was done by performing numerical simulation of a linear stroke MR damper operating in different modes and comparing the results of computations with the results of experimental investigation of an analogical MR damper described in [31]. A series of steady state flow analyses of the hydrodynamic pressure affecting the piston of the MR damper, resulting in its resistance force, at a different speed of piston movement, as well as different strength of magnetic field, were performed. The viscosity consistency *K* was being adjusted until the results of the computer simulation reached a satisfactory conformance with the experimentally obtained resistance force (difference less than 5%).

**Figure 10:**
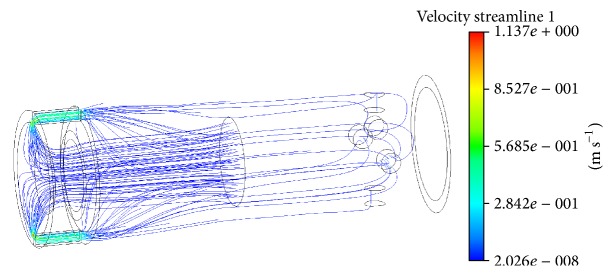
Trajectories and velocities of the magnetorheological fluid flow in MR damper in the case of flow input velocity corresponding to 0.0075 m/s piston speed and 25 mT magnetic field.

**Figure 11:**
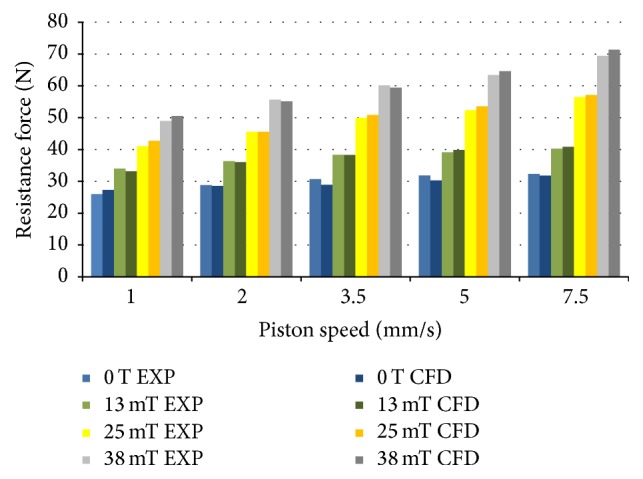
Comparison of experimentally determined (EXP) and calculated (CFD) resistance forces generated by a MR damper at different speeds of the piston and different magnetic field strength (0 T; 13 mT; 25 mT; and 38 mT magnetic field).

Figure 10 shows the trajectories and velocities of magnetorheological fluid flow in a MR damper when the flow input velocity corresponds to 0.0075 m/s speed of the piston and 25 mT magnetic field strength. The final values of yield stresses *τ*_*Y*_ and viscosity consistency *K*, used for the description of the MR fluid MRF140-CG as non-Newtonian by means of Herschel-Bulkley mathematical model, are given in Table 2. Comparison of experimentally determined and calculated resistance forces generated by a MR damper at different speeds of the piston and different magnetic field strength is shown in Figure 11.

### 4.2. Magnetorheological Resistive Exercise Device for Rowing Machine

The virtual prototype of the twin-tube linear motion magnetorheological resistive exercise device for a lever-type rowing machine was elaborated according to the structural scheme of such a device described in Section 3.3 (Figure 8). A parametrical 3D geometrical model of the magnetorheological resistive exercise device characterized by the following structural features was created by using of 3D CAD system SolidWorks:

- Body scheme: twin tube;
- Piston stroke: 280 mm;
- Diameter of the piston (inner tube internal diameter): 27 mm;
- Number of channels for fluid passage between the compression and tubular chambers of cylinder: 5;
- Diameter of the channels in the bushing for fluid passage between the chambers of cylinder: 2 mm;
- Length of the base valve bushing: 17 mm.

**Figure 12:**
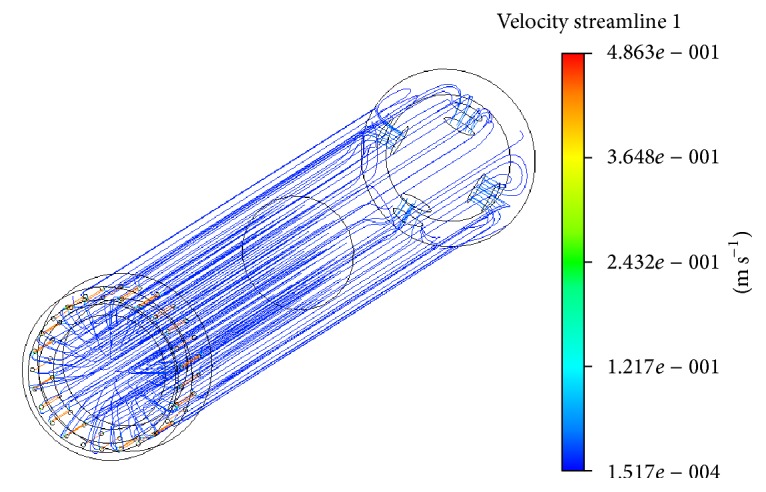
Trajectories and velocities of magnetorheological fluid flow in MR resistive exercise device for rowing machine (number of the channels in the base valve bushing for fluid passage between the chambers: 25; diameter: 0.8 mm).

On the basis of the 3D geometrical model of a MR resistive exercise device, the computational model of its cavities filled with MR fluid MRF140-CG (the same as in case of MR damper) was built. Computations of resistance force generated by the MR resistive exercise device at different working modes (strength of magnetic field and piston speed) were carried out by means of ANSYS CFX CFD software (Figure 12) in analogy with numerical simulation of a MR damper (Section 4.1). During this numerical simulation the MR fluid non-Newtonian properties (dependent on magnetic field strength; see Table 2) obtained during the previous stage of the research were taken into account.

The numerical modeling of the MR resistive exercise device had a double purpose. First of all, the number and cross-sectional area (or diameter) of the base valve bushing (pos. 6, Figure 8) channels CH (Figure 8) for the MR fluid passage between the compression and tubular chambers of the device (CC and TC, Figure 8) had to be clarified, ensuring the minimal resistance (dozens of newtons, Section 2.2) force during the recovery phase (passive mode or 0 T magnetic field strength). Afterwards, the number and cross-sectional area (or diameter) of these channels ensuring maximal resistance force during drive phase of the rowing stroke cycle had to be figured out. This force should be near 5500 N in the case of 1000 N force on the handle of a L-shaped lever imitating the oar and the ratio of its arms *L*_1_ and *L*_2_ equal to 5.5 (Figure 1 and Section 2.2). The piston speed was near maximal (0.3 m/s) in both cases, while the maximal magnetic field strength for the drive phase was equal to 38 mT. Simulation was carried out by increasing the number of the channels from 5 to 30 with a step of 5 and reducing the diameter of the channels from 2 mm to 0.4 mm every 0.4 mm.

Computations have shown that the minimal (for the recovery phase) resistance force resulting from the hydrodynamic pressure acting on the piston is ensured even when the base valve bushing contains 5 or 10 channels of 1.2–2.0 mm diameter. However, in such a case the necessary maximal resistance force in the drive phase is not reached even when the maximal strength magnetic field (38 mT) is applied (the base valve is too leaky). As it is shown below, the necessary range of the resistance force (0–5500 N) was obtained when the number of the channels was increased to 25 and their diameter reduced to 0.8 mm.

Supplementary analysis was carried out to verify the final design (base valve with 25 channels of 0.8 mm diameter) and the computational model of the MR resistive exercise device. The resistance force generated by a device with such parameters operating in a passive mode (with no effect of the magnetic field on MR fluid viscosity) was calculated and compared with the results of experimental research of regular hydraulic cylinder number 2 (having the same diameter piston) used in a rowing machine [27]. It was found that the calculated values of the resistance force (at different piston speeds) differ from those obtained experimentally less than 2.5%. Consequently, it was stated that the number and diameter of the channels are selected properly and the computational model may be used for further simulations.

### 4.3. Control of the Resistance Force within Rowing Stroke by Changing Magnetic Field Acting the MR Fluid

The last two series of numerical analyses were carried out to determine the law of change of the magnetic field strength directly influencing MR fluid viscosity and thereby the resistance force, generated by the magnetorheological resistive device, which would ensure the pattern of the resistance force on the oar handle adequate for real rowing.

The first set of computations (using models of MR fluid and the magnetorheological resistive device described above) gave the dependencies of the resistance force generated by the device on the piston speed and magnetic field strength. It was found out that these dependencies are practically linear; the maximal resistance force when the piston speed is maximal (0.3 m/s) is near 1000 N in a passive mode and 2000, 4000, and 5550 N when the strength of the magnetic field applied is 13, 25, and 38 mT, respectively. It means that the maximal hydrodynamic force acting on the piston, that is, the resistance force of a MR device, is 5550 N (at the maximal piston speed and maximal strength magnetic field). If such a device were installed into the rowing machine with the ratio of the L-shaped lever arms *L*_1_ and *L*_2_, as shown in Figure 1, the force acting on the oar handle would be 5.5 times lower, about 1000 N. This force exceeds the maximal force (600–700 N), which is usually reached during the real rowing; nevertheless, such a reserve of the maximal force can be useful, for example, when it is necessary to compensate the change of MR fluid properties (decrement of viscosity) due to its heating after a large number of exercising cycles or simply to reduce the heating intensity due to training at a reduced load.

**Figure 13:**
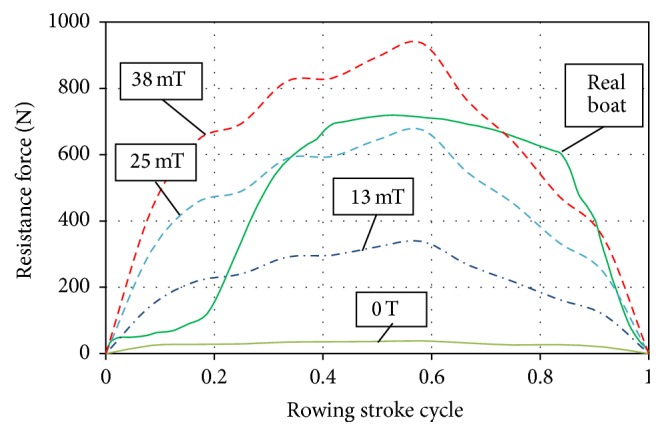
The patterns of resistance force on the oar handle when rowing a real boat [20] and when exercising on a rowing machine with MR loading device (at strength of the magnetic field (0 T, 13 mT, 25 mT, and 38 mT)).

One more series of computations was carried out in order to find out how the strength of the magnetic field generated by the coil of a MR resistive exercise device should be changed within a single stroke cycle to ensure the pattern of the resistance force adequate for the force on the oar of a real boat. In this case, the size of this force at different moments of a normalized rowing stroke cycle was calculated by taking into account the variation of piston speed during the stroke and the effect of a different strength magnetic field, which was simulated by a corresponding set of parameters describing the properties of the MR fluid. It may be noticed that the force pattern of rowing a real boat [20] differs significantly from the force patterns given by the exerciser with MR resistive exercise devices when the strength of the magnetic field which acts on the MR fluid is constant (0 mT, 13 mT, 25 mT, and 38 mT, Figure 13). In the case of minimal strength of the magnetic field (and correspondingly viscosity of the MR fluid), the curve of resistance force generated by the device lies substantially below the representative force curve of real rowing (Figure 13, curves 0 mT and 13 mT), but when the magnetic field strength is increased, the resistance force grows up, and, in the case of the strongest magnetic field, it exceeds the necessary level (Figure 13, curve 38 mT). This fact suggests the conclusion that the force generated by a MR resistive exercise device may be controlled by a corresponding change of strength of the magnetic field affecting MR fluid at the relevant time moments of the exercise stroke, and such control may give the pattern of resistance force reconstructing the variation of resistance force adequate for the force on the oar resulting when rowing a real boat.

**Figure 14:**
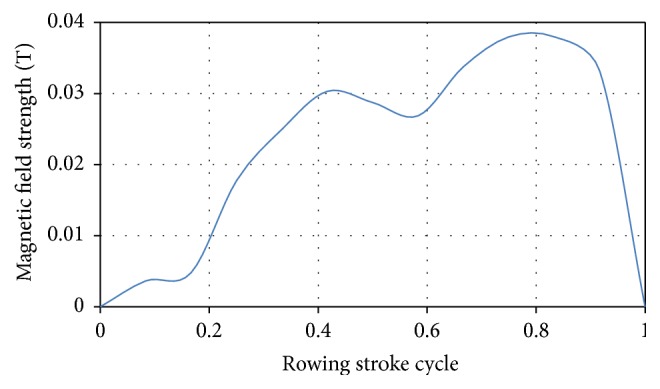
Law of variation of magnetic field strength giving the pattern of resistance force corresponding to rowing of the real boat.

The law of variation of the magnetic field strength generated by the coil of a MR resistive exercise device of a lever-type rowing machine giving the pattern of resistance force within a single stroke cycle adequate for rowing a real boat (curve “real boat” in Figure 13) at 20°C temperature is shown in Figure 14. It was obtained by performing computations of resistance force generated by the controllable resistance device during which normalized reference curves of the piston speed and resistance variation during the stroke were combined as described in Section 3.4.

**Figure 15:**
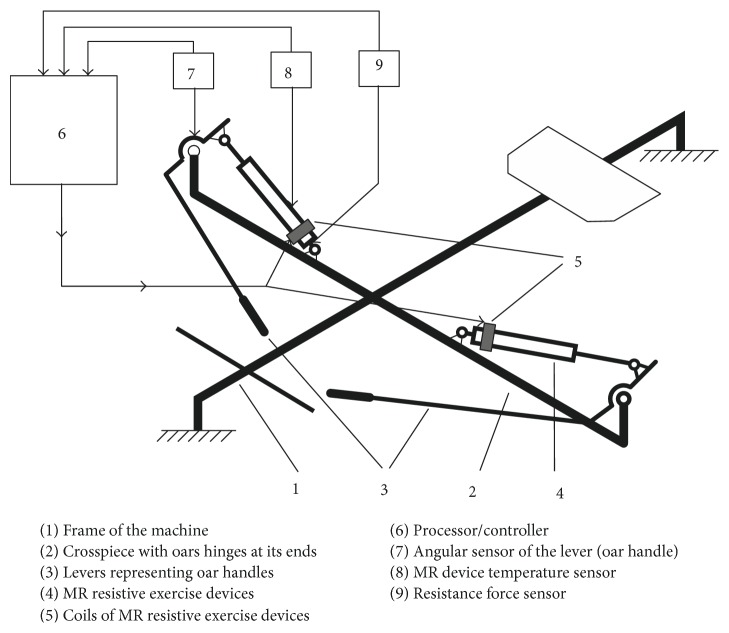
Scheme of lever-type rowing machine with the programmable control system of MR resistive exercise device.

To ensure the pattern of resistance force corresponding to rowing a real boat, a rowing machine should be equipped with a programmable control system with a set of sensors measuring various kinematic-dynamic parameters during the exercise cycle, such as angular sensors of the vertical and horizontal position of levers simulating oars, resistance force, and temperature sensors (Figure 15). In this way, the strength of the electric current in the coils of MR resistive exercise devices could be controlled by the processor/controller according to the position and speed of movement of the oars, temperature of hydraulic cylinders, or even biomechanical and physiological parameters of the athlete. Thus, there would be a possibility to simulate adequately the variation of rowing force during a single stroke and a series of strokes (training session or competition) or even to use an exerciser for rehabilitation needs (by ensuring isotonic or isokinetic modes of training) or simply to compensate reduction of the resistance force due to heating of the device during the training session.

## 5. Conclusions

Improvement of techniques and learning of psychomotor skills helping professional athletes to achieve maximal sports performance is possible only in cases when exercisers used by them conform to the kinematics of movements and the resistance typical for particular sports. However, most available rowing simulators are suitable only for training the general physical condition because usually they are of specific kinematics and their resistance characteristics are not adequate for real rowing, unstable, and practically uncontrollable.

The magnetorheological resistive exercise device and its control system for a lever-type rowing machine have been developed that can ensure variation of resistance force on the oar handle adequate for hydrodynamic resistance which occurs during a real boat rowing stroke. Computational fluid dynamic simulations of the device have been carried out to employ the magnetorheological fluid technology for solving the resistance force control problem. To find the main structural parameters of a device giving maximal adequacy of the resistance to real rowing, the MR fluid circulating in the device was modeled as non-Newtonian. To that purpose, the dependencies of parameters describing properties of magnetorheological fluid MRF140-CG on the strength of the magnetic field used for the control of resistance force generated by the device were found and used during the numerical modeling. After verification of the computational model, the law of variation of the magnetic field strength was figured out, giving the pattern of resistance force corresponding to rowing a real boat.

The possibility of controlling resistance during a single stroke also ensures simulating sequences of different resistance strokes in the case of reconstruction of a training session or competition and using the exerciser for rehabilitation needs (by ensuring isotonic or isokinetic modes of training). Furthermore, the controllable resistive device and results of magnetorheological fluid numerical modeling presented in this paper may also be employed in development of other sports and rehabilitation equipment based on magnetorheological technology.

## Figures and Tables

**Table 1:** Main physical properties of MR fluid MRF-140CG.

Physical property	Newtonian	Non-Newtonian
Density, kg/m^3^	3640	3640
Specific heat, J/(kg·K)	800	800
Thermal conductivity, W/(m·K)	2.3	2.3
Dynamical viscosity, Pa·s	0.28	According to Herschel-Bulkley model
Power law index	—	0.8
Minimal shear strain rate, 1/s	—	100
Maximal shear strain rate, 1/s	—	800
Time constant, s	—	5

**Table 2:** Values of yield stresses *τ*_*Y*_ and viscosity consistency *K* of MR fluid at different strength of magnetic field *B*.

*B*, mT	*τ*_*Y*_, Pa	*K*, Pa
13	800	5
25	1600	14
38	2600	18
